# Free Radical Scavenging Activity of Carbonyl-Amine Adducts Formed in Soybean Oil Fortified with Phosphatidylethanolamine

**DOI:** 10.3390/molecules25020373

**Published:** 2020-01-16

**Authors:** Jana Goritschnig, Klaudia Tadus, Jürgen König, Marc Pignitter

**Affiliations:** 1 Department of Physiological Chemistry, Faculty of Chemistry, University of Vienna, Vienna 1090, Austria; a01168730@unet.univie.ac.at (J.G.); klaudia.t@hotmail.com (K.T.); 2 Department of Nutritional Science, Faculty of Life Sciences, University of Vienna, Vienna 1090, Austria; juergen.koenig@univie.ac.at

**Keywords:** phosphatidylethanolamine, soybean oil, lipid oxidation, hexanal, radical scavenging activity, carbonyl-amine adducts

## Abstract

Non-enzymatic browning reactions between lipid aldehydes and aminophospholipids might play an important role in the oxidative stability of cold-pressed vegetable oils. We, therefore, aimed to study the Maillard-type reaction between hexanal, a lipid oxidation product of linoleic acid, and phosphatidylethanolamine (PE (16:0/18:1)) at a ratio of 2:1 at conditions representative of the extraction of cold-pressed soybean oils (CPSBO) and determine the radical scavenging activity of the carbonyl-amine adducts with the 2,2-diphenyl-1-picrylhydrazyl (DPPH) assay. The reaction product, 2-pentyl-3,5-dibutyl-dihydropyridine, could be identified by means of LC-ESI-QTOF-MS/MS. The formation of this nitrogen-containing heterocycle significantly increased with time and temperature (*p* < 0.05). The products formed during the carbonyl-amine reaction between PE (16:0/18:1) and hexanal at 60 °C showed a radical scavenging activity of approximately 20% (*p* < 0.05). The fraction, containing 2-pentyl-3,5-dibutyl-dihydropyridine, contributed to, but was not solely responsible for, the radical scavenging activity (*p* < 0.05). Incubation of CPSBO fortified with PE (16:0/18:1) at 60 °C for 60 min had the strongest radical scavenging activity of 85.1 ± 0.62%. Besides 2-pentyl-3,5-dibutyl-dihydropyridine, other carbonyl-amine adducts might impact the radical scavenging activity of CPSBO as well. The oxidative stability of CPSBO might be increased by promoting the formation of carbonyl-amine reaction products, such as 2-pentyl-3,5-dibutyl-dihydropyridine.

## 1. Introduction

Many cold-pressed vegetable oils are rich in polyunsaturated fatty acids (PUFAs), which are important components of a balanced diet. Replacing saturated fatty acids (SFAs) with PUFAs has been linked to a reduction in cardiovascular events [1]. In addition, the replacement of carbohydrates and SFAs with PUFAs was shown to improve certain parameters of the blood sugar regulation [2]. However, PUFA-rich oils lack oxidative stability, thus generating lipid oxidation products, such as aldehydes, which have been linked to adverse health effects [3]. Hence, it is of utmost importance to identify strategies which help to enhance the oxidative stability of these oils by trapping lipid radicals as well as inhibiting the formation of lipid aldehydes [4,5].

Soybean oil is rich in PUFAs, with linoleic acid (LA) being the most abundant fatty acid, contributing to more than half of the total fatty acid composition [6]. Thermal oxidation of soybean oil at 60 °C for 60 min led to the accumulation of volatile compounds, such as hexanal at a relative concentration of 23.5%, which was the second most prevalent volatile in the oxidized soybean oil, with a peroxide value of five [7]. Hexanal was mainly formed as a secondary oxidation product through the decomposition of 13-hydroperoxides during the autoxidation of LA [8]. In fact, the hexanal content of soybean oil increased in relation to its LA content from 0.1 ± 0.01 to 0.53 ± 0.02 ng/mg LA during eight weeks of storage at household conditions [9]. An increased content of volatile compounds, such as aldehydes, in vegetable oils has been associated with a decreased acceptance of these oils [10]. Furthermore, the hexanal content was reported to be an indicator for the oil quality and is able to predict the sensory quality of soybean oil [11].

Crude soybean oil contains various minor compounds, such as tocopherols, chlorophyll, free fatty acids, sterols, metals or phospholipids (PLs) [12,13]. The phosphorous content in soybean oil was shown to be higher than in various other vegetable oils, such as corn or palm oil. However, in refined oils, most PLs were lost during the degumming process [13]. In contrast, crude soybean oil is still rich in PLs [14]. The most prevalent PL classes in crude soybean oil are phosphatidylcholine (PC), phosphatidylethanolamine (PE) and phosphatidylinositol (PI). PE contributes to about one fifth to one third of the total PL content in crude soybean oil [14,15,16]. PLs frequently have a SFA on their sn-1 position and an unsaturated fatty acid on their sn-2 position [17]. 1-Palmitoyl-2-oleoyl-sn-glycero-3-phosphoethanolamine (PE (16:0/18:1)) has already been identified as one of the three main molecular species of PE in soybeans, contributing to approximately 10% of the total PE content [18]. The fortification of oils with PL have been shown to have both prooxidative and antioxidative effects [19,20,21,22]. There are different theories, why PLs might have antioxidative properties [22], such as synergism with tocopherol [19,23,24], chelation of prooxidative metals [25,26] or Maillard-type reactions between PE and lipid aldehydes [27,28].

The Maillard reaction usually refers to the reaction between the amino group of an amino acid or peptide with the carbonyl group of a reducing sugar, leading to a large variety of reaction products through various reaction pathways. However, lipid oxidation and the Maillard reaction should not be considered as two completely separate mechanisms [29]. In fact, the formation of Maillard reaction products, such as pyrroles, and browning development was observed in the reaction between the amino group of PE and ribose as well as in the traditional Maillard reaction between lysine and ribose [30]. Furthermore, lipid aldehydes have the ability to form Maillard reaction products with PE as well [31,32]. Maillard reaction products were shown to have antioxidant properties [33,34], which have been associated with the browning development [33,34]. Non-enzymatic browning reactions between lipid aldehydes and PE might play an important role in the oxidative stability of vegetable oils as well [27,28,35]. However, studies characterizing the molecular structure and antioxidant activity of carbonyl-amine adducts in cold pressed oils are lacking. Therefore, we aimed to study the Maillard-type reaction between hexanal, a lipid oxidation product of LA, and PE (16:0/18:1) at conditions representative of the extraction of cold pressed oils [36] to find strategies that possibly enhance the oxidative stability of cold pressed oils.

## 2. Results and Discussion

### 2.1. Tentative Identification of a Carbonyl-Amine Adduct by LC-ESI-QTOF-MS/MS

**Figure 1:**
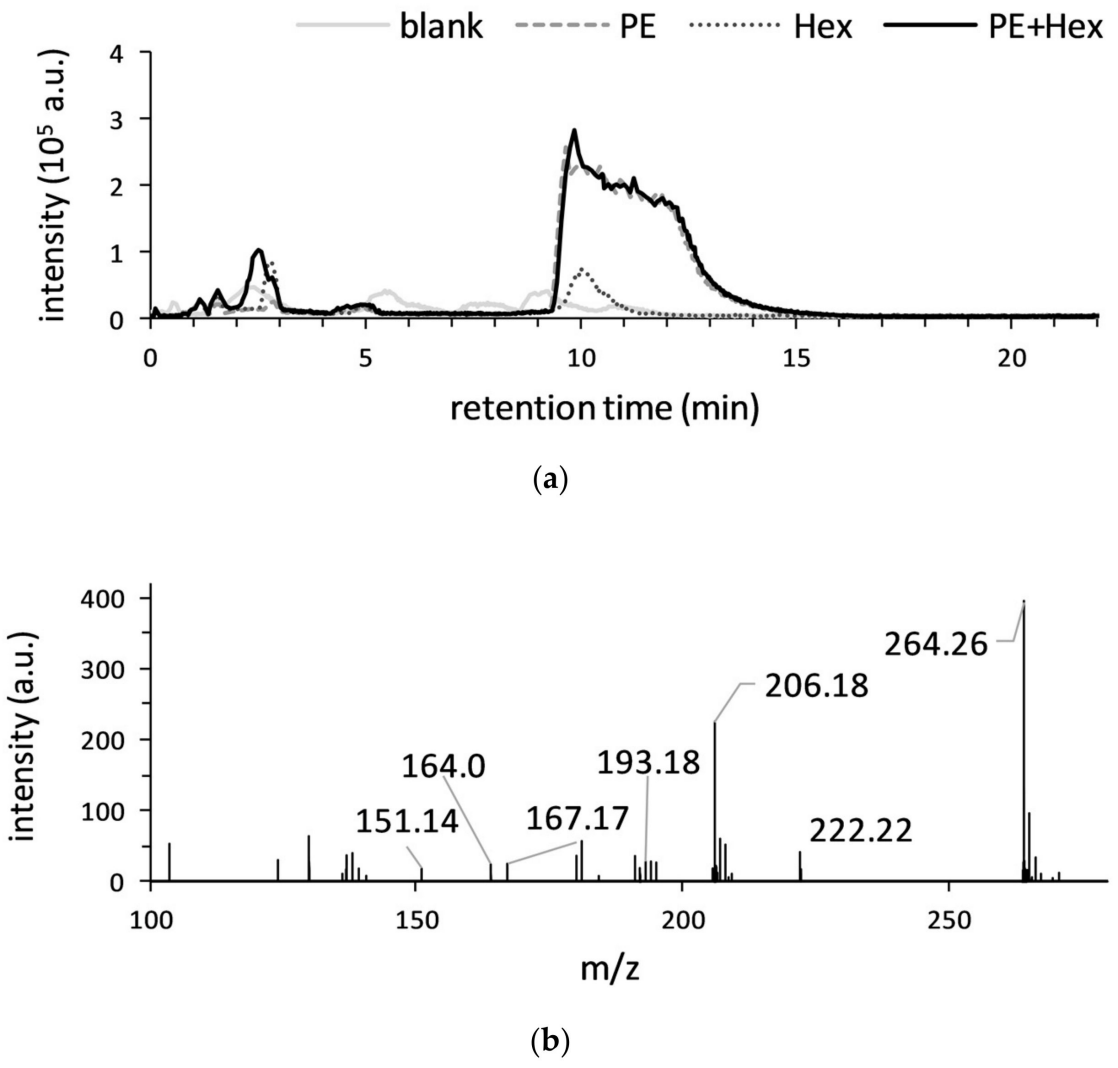
Tentative identification of a carbonyl-amine adduct derived from phosphatidylethanolamine (PE) (16:0/18:1) and hexanal. Overlaid base peak chromatograms (*m/z* 50–2500) obtained from LC-ESI-QTOF-MS in positive ion mode (**a**). PE (16:0/18:1) (0.8 mM) and hexanal (1.6 mM) were incubated in the dark at 40 °C for 21 h. LC-ESI-QTOF-MS/MS spectrum of *m/z* 264.26 was obtained in positive ion mode with the collision energy of 15 eV (**b**). PE: PE (16:0/18:1); Hex: hexanal.

**Scheme 1:**
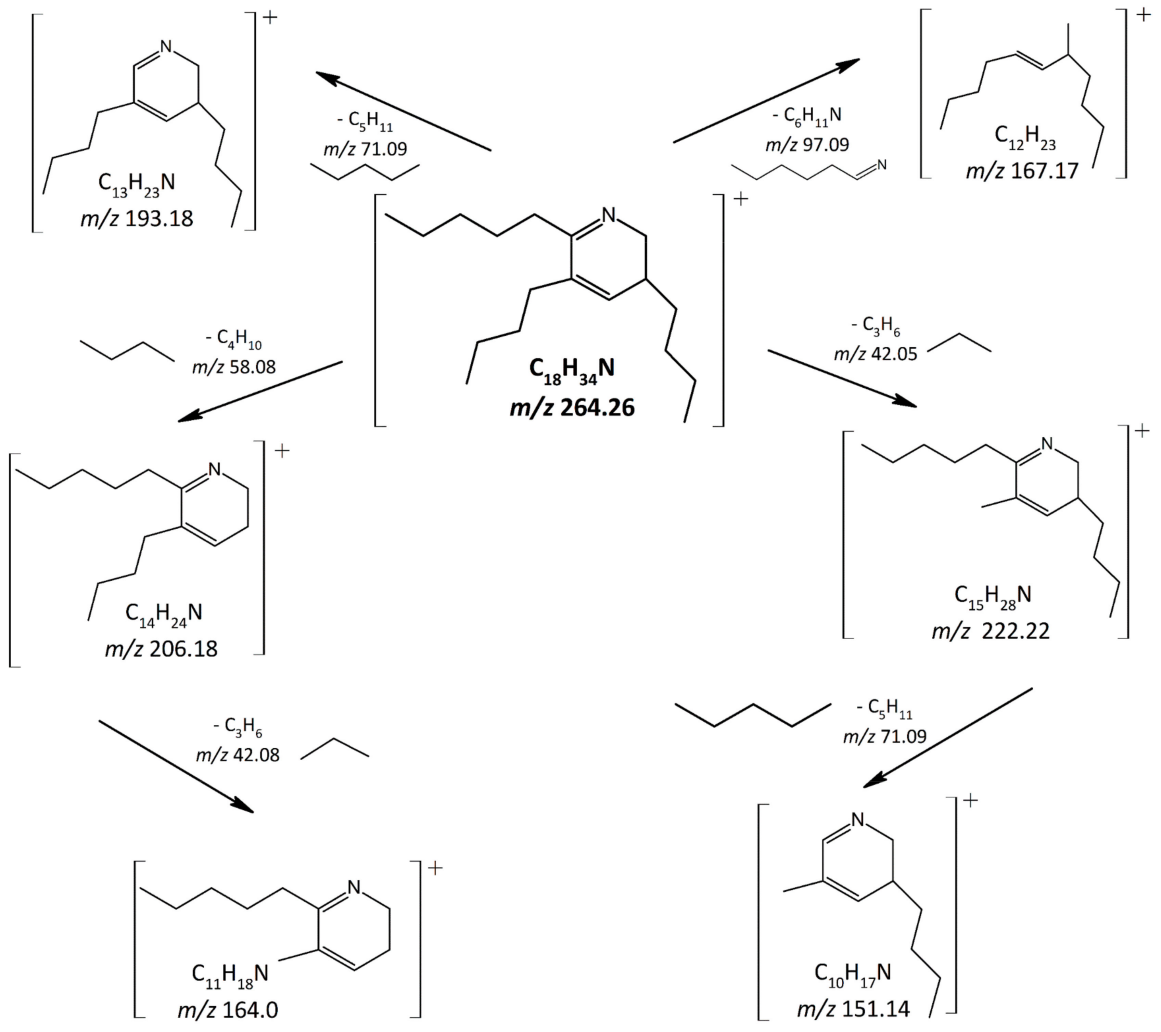
The proposed MS fragmentation pathway of 2-pentyl-3,5-dibutyl-dihydropyridine.

The incubation of PE (16:0/18:1) with hexanal resulted in the formation of a carbonyl-amine adduct (*m/z* 264.26) eluting at 2.5 min, as shown in the base peak chromatogram in Figure 1a. No peaks were detected at *m/z* 264.26 in hexanal or PE (16:0/18:1) alone. The mixture of PE (16:0/18:1) and hexanal was incubated overnight (21 h, 40 °C) to achieve an adequate yield for the tentative identification. To not only rely on the measured mass and to reduce the number of possible molecular formulas, MS/MS experiments were performed. Figure 1b shows the MS/MS spectrum of the carbonyl-amine adduct at *m/z* 264.26 corresponding to the theoretical *m/z* of C_18_H_34_N in positive ion mode. For the tentative identification of the carbonyl-amine adduct, the fragments of the MS/MS spectra were assigned to the proposed chemical structures. These fragments led to the tentative identification of 2-pentyl-3,5-dibutyl-dihydropyridine as a potential reaction product. Scheme 1 shows the proposed MS/MS fragmentation pathway of the identified compound. The peak at *m/z* 222.22 corresponds to the loss of a C_3_H_6_ group (*m/z* 42.05) through σ-scission and *m/z* 151.14 corresponds to a further loss of an alkyl group (C_5_H_11_ with *m/z* 71.09) through α-scission. The peak at *m/z* 206.18 represents a σ-scission of C_4_H_10_ (*m/z* 58.08) and *m/z* 164.0 appears after a further loss of C_3_H_6_ (*m/z* 42.08) through σ-scission. Similarly, α-scission of C_5_H_11_ (*m/z* 71.09) led to the generation of *m/z* 193.18. A ring opening reaction followed by α-scission of C_6_H_11_N (*m/z* 97.09) induced the formation of *m/z* 167.17. Hwang et al. [37] identified 2-pentyl-3,5-dibutyl-dihydropyridine as a reaction product of hexanal and ammonium sulfide at room temperature applying mass spectrometry. MS fragments obtained were similar to those in the current study, further confirming the identity of the carbonyl-amine adduct. In addition to the MS results, NMR spectra were recorded of the fraction, isolated from the mixture of PE (16:0/18:1) and hexanal. ^1^H NMR (δ, 500 MHz, CDCl_3_): 0.88 (9H, t, J = 6.71 Hz, CH_3_-CH_2_-CH_2_), 2.03 (2H, q, J = 7.29 Hz, CH_2_-CH_2_-C_pyr_H), 2.23 (1H, m, J = 7.56, 5.08 Hz, C_pyr_H_2_-C_pyr_H-CH_2_), 2.35 (4H, t, J = 7.47 Hz, C_pyr_H-CH_2_-CH_2_), 3.39 (1H, t, J = 7.12 Hz, N-C_pyr_H-CH_2_), 5.78 (1H, d, J = 3.73 Hz, C_pyr_H-C_pyr_-C_pyr_H), 8.00 (1H, s, N-C_pyr_H-C_pyr_). However, to the best of our knowledge, this carbonyl-amine adduct has not been described as a reaction product between PE and hexanal. 

**Scheme 2:**
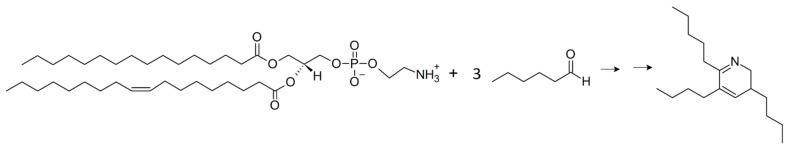
The proposed formation of 2-pentyl-3,5-dibutyl-dihydropyridine.

The incubation of PE with aldehydes led to various reaction products, of which only a few had already been identified. The formation of non-cyclic [31,38] and cyclic adducts [32,39], such as pyrroles and pyridines, have already been observed in reactions between PE and aldehydes. The formation of non-cyclic carbonyl-amine adducts between PE (18:1/18:1) (0.8mM) and heptadecanal (1.48 mM) was observed during incubation at 37 °C for 2 h by Standelmann-Ingrand et al. [38]. However, the formation of cyclic adducts was not investigated in their study. In the current study, the incubation of PE (16:0/18:1) and hexanal under similar conditions led to the formation of a cyclic carbonyl-amine adduct. Annibal et al. [39] analyzed the formation of various reaction products including a pyridine adduct by the reaction between 1,2-dipalmitoyl-sn-glycero-3-PE (PE (16:0/16:0)) and hexanal in water. The conjugation of three hexanal molecules to the amino group of PE (16:0/16:0) resulted in the formation of a pyridine ring with a PE (16:0/16:0) sidechain at *m/z* 936. Due to the structural similarities with the present reaction product, it can be proposed that in the current study, three hexanal molecules might have reacted with the head-group from PE (16:0/18:1) (Scheme 2). However, incubation in a lipophilic media, such as chloroform-methanol (2:1), led to the loss of major parts of PE (16:0/18:1) and to the formation of the dihydropyridine adduct (*m/z* 264.26) without an intact PL sidechain. Differences in the polarity of the solvents might contribute to the observed effect. PLs are amphiphilic and are known for their ability to self-assemble into colloidal structures in aqueous media [40]. Therefore, the formation of colloidal structures might be responsible for the protection of fatty acids in the PL-structure during the incubation in water. In lipophilic media, PLs were shown to be able to form reverse micelles, when incubated over the critical micelle concentration in the presence of small amounts of water (<0.3% *w/w*) [41], possibly explaining the formation of 2-pentyl-3,5-dibutyl-dihydropyridine without an intact PL sidechain in the current study. In reverse micelles, the acyl chains are not protected in the micelle core, thereby the cleavage of acyl chain might be feasible. However, it might also be conceivable that the product of the aldolic condensation of two hexanal molecules reacts with the aminophospholipid to form an α,β-unsaturated aldimine. The addition of a third hexanal molecule to the aldimine leads to ring closure and the formation of a dihydropyridine, which might, although not observed in the current study, even be converted to a pyridine under oxidative conditions [42].

### 2.2. Time- and Temperature-Dependent Formation of the Carbonyl-Amine Adduct

**Figure 2:**
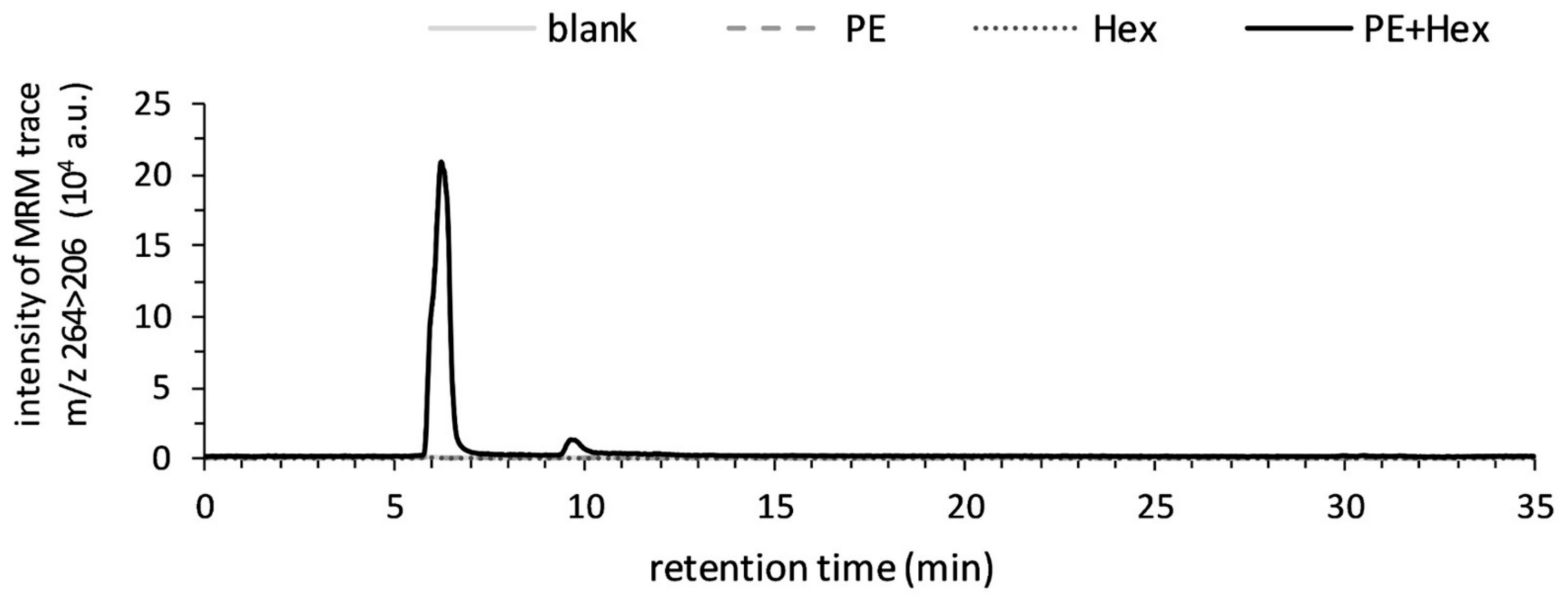
Formation of 2-pentyl-3,5-dibutyl-dihydropyridine in the presence of both, PE (16:0/18:1) (0.8 mM) and hexanal (1.6 mM). Overlaid chromatograms (MRM: *m/z* 264 > 206) recorded with LC-MS in ESI-positive ion mode. Samples were incubated in the dark at 60 °C for 60 min.

**Figure 3:**
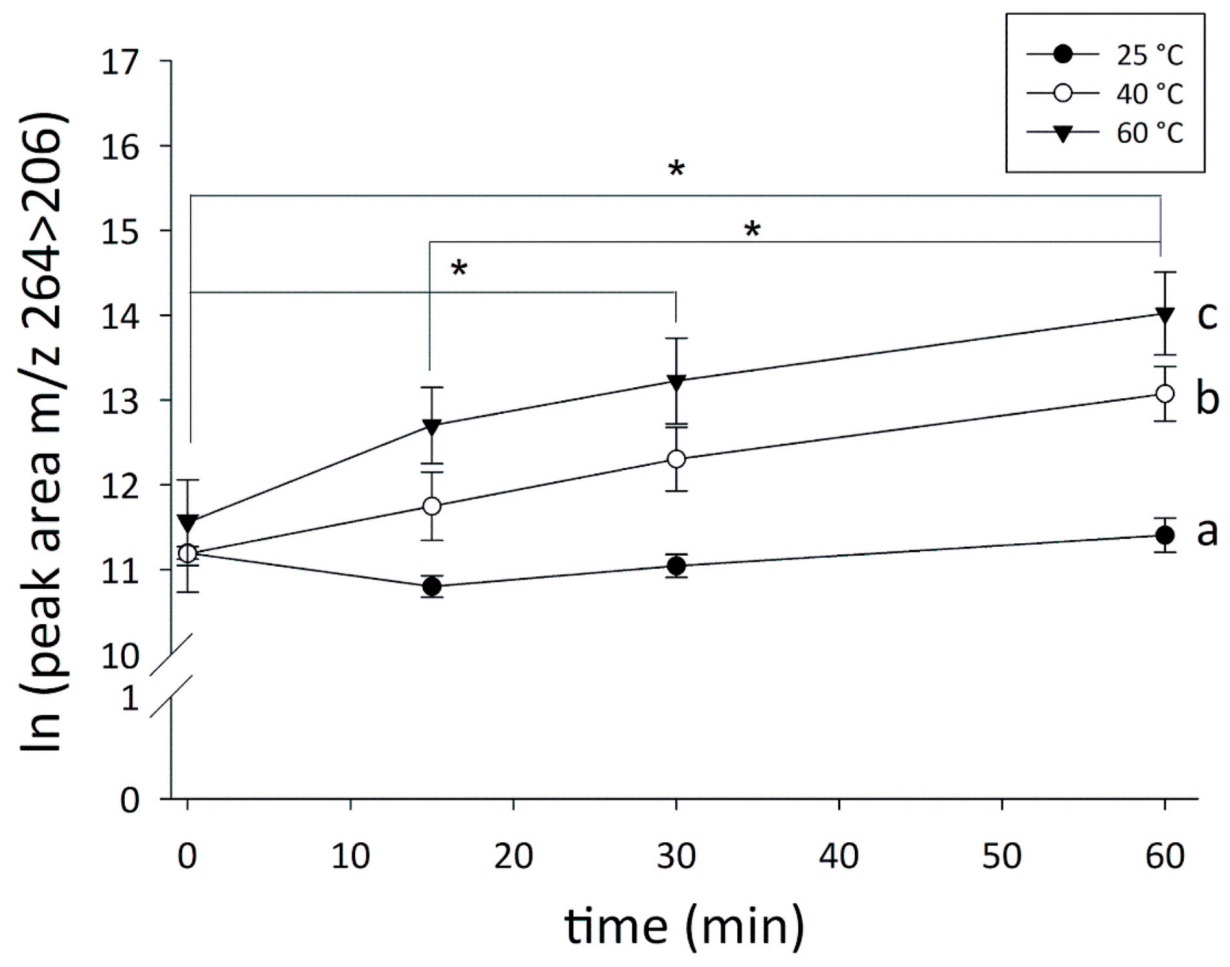
Time- and temperature-dependent formation of 2-pentyl-3,5-dibutyl-dihydropyridine. The mixture of PE (16:0/18:1) (0.8 mM) and hexanal (1.6 mM) was incubated at 25, 40 and 60 °C for 0, 15, 30 and 60 min. After natural log-transformation to obtain a normal distribution, two-way analysis of variance (ANOVA) with the Holm—Sidak method was applied (*p <* 0.05). Significant differences in time are presented with asterisks and differences between temperatures are expressed with different letters. Values (*n* = 5) are presented as mean ± SEM. Filled circle: 25 °C; circle: 40 °C, filled triangle: 60 °C.

After the LC-MS/MS identification, the time- and temperature-dependent formation of 2-pentyl-3,5-dibutyl-dihydropyridine was investigated. Therefore, the mixture of PE (16:0/18:1) and hexanal in chloroform-methanol (2:1, *v/v*) was incubated at 25, 40 and 60 °C for 0, 15, 30 and 60 min and analyzed by LC-MS. The samples were recorded using positive ionization in multiple reaction monitoring mode (MRM) and the peak at *m/z* 264 > 206 was used for the comparative analysis. The overlaid chromatograms of the samples incubated at 60 °C for 60 min in Figure 2 show that the 2-pentyl-3,5-dibutyl-dihydropyridine was present in the mixture of PE (16:0/18:1) and hexanal and absent in PE (16:0/18:1) or hexanal alone. Elevating the incubation time (*p* < 0.001) and temperature (*p* < 0.001) increased the formation of the dihydropyridine significantly, as shown by the natural log-transformed results in Figure 3. Thus, the carbonyl-amine adduct might be formed at temperatures that can be reached during the mechanical extraction of cold-pressed oil [36].

### 2.3. Estimation of Radical Scavenging Activity of PE (16:0/18:1) and Hexanal

**Figure 4:**
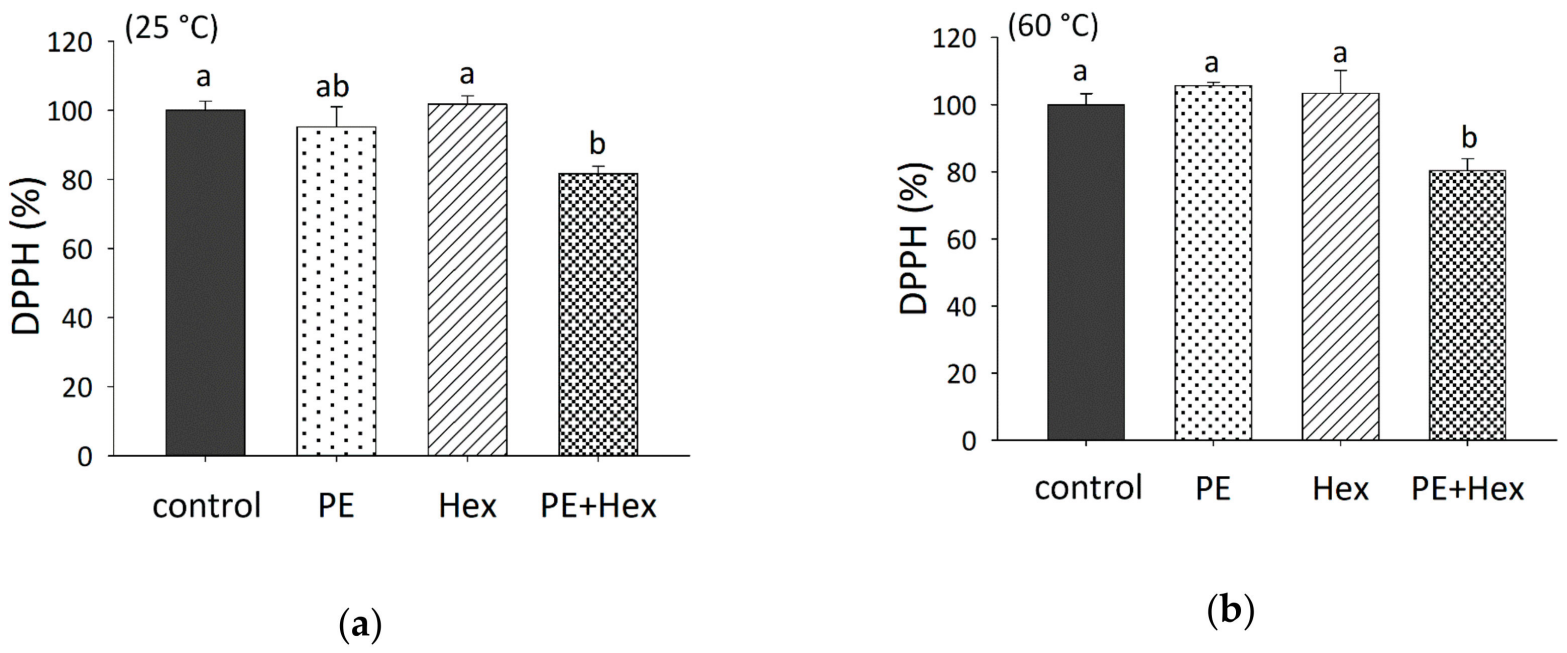
Free radical scavenging activity of PE (16:0/18:1) (0.5 mM) and/or hexanal (1 mM) as determined by the 2,2-diphenyl-1-picrylhydrazyl (DPPH) assay in toluene. Samples and control, which consisted of chloroform-methanol (2:1), were pre-treated for 60 min either at 25 (**a**) or 60 °C (**b**), followed by incubation with DPPH for 60 min. The DPPH absorbance (520 nm) in the samples (*n* = 3) was compared to the control, which was set to 100%. Each experiment was performed thrice. Different letters indicate significant differences. Statistical analysis was performed by applying one-way ANOVA with the Holm–Sidak post hoc test.

To estimate the potential antioxidant activity of such carbonyl-amine adducts, the radical scavenging activity of the samples, which were incubated at 25 and 60 °C for 60 min, was determined with the DPPH assay. At 25 °C, the mixture of PE (16:0/18:1) and hexanal significantly increased the radical scavenging activity by approximately 20%, compared with hexanal or the control (chloroform-methanol) (Figure 4a).

At 60 °C, the radical scavenging activity of PE (16:0/18:1) with hexanal (19.6 ± 3.48%) was significantly higher than the radical scavenging activity of chloroform-methanol alone (0.00 ± 3.25%), hexanal (−3.36 ± 6.90%) or PE (16:0/18:1) (−5.69 ± 1.03%) (Figure 4b). These results indicate a significant radical scavenging effect due to the presence of carbonyl-amine adducts, whereas the signal intensity of DPPH remained unchanged after incubation with PE (16:0/18:1) or hexanal alone. However, it has to be noted that the free radical scavenging activity of the carbonyl-amine adducts formed by PE and hexanal is weak, since other antioxidants, such as γ-tocopherol, were shown to increase the radical scavenging activity by more than 80% at a concentration of only 50 µM [9]. 

Although the LC-MS/MS results confirmed that the incubation of PE (16:0/18:1) and hexanal at 60 °C for 60 min resulted in a slightly more pronounced formation of 2-pentyl-3,5-dibutyl-dihydropyridine than at 25 °C, no significant differences between the radical scavenging activity of PE (16:0/18:1) with hexanal at 25 and 60 °C could be obtained. The concentration of the identified carbonyl-amine adduct might be too low to contribute to any significant effects between both temperatures but high enough to induce significant effects within each temperature group. Hidalgo et al. [35] reported that the fortification of refined olive oil with PE (200–400 ppm) resulted in the formation of carbonyl-amine adducts such as pyrroles and that the addition of PE delayed the induction period of lipid oxidation. However, at a lower concentration of PE (100 ppm) no effects were observed [35]. These results indicate that carbonyl-amine adducts contribute to the antioxidant activity depending on their concentration. The initial concentration of PE as well as the incubation time and temperature might influence whether and to which extent these carbonyl-amine adducts are formed, although in the current study the slight increase of 2-pentyl-3,5-dibutyl-dihydropyridine by approximately 23% at 60 °C compared to 25 °C after 60 min was not sufficient to affect the radical scavenging activity of the carbonyl-amine adduct.

### 2.4. Estimation of Radical Scavenging Activity of the Fraction Containing Carbonyl-Amine Adducts

**Figure 5:**
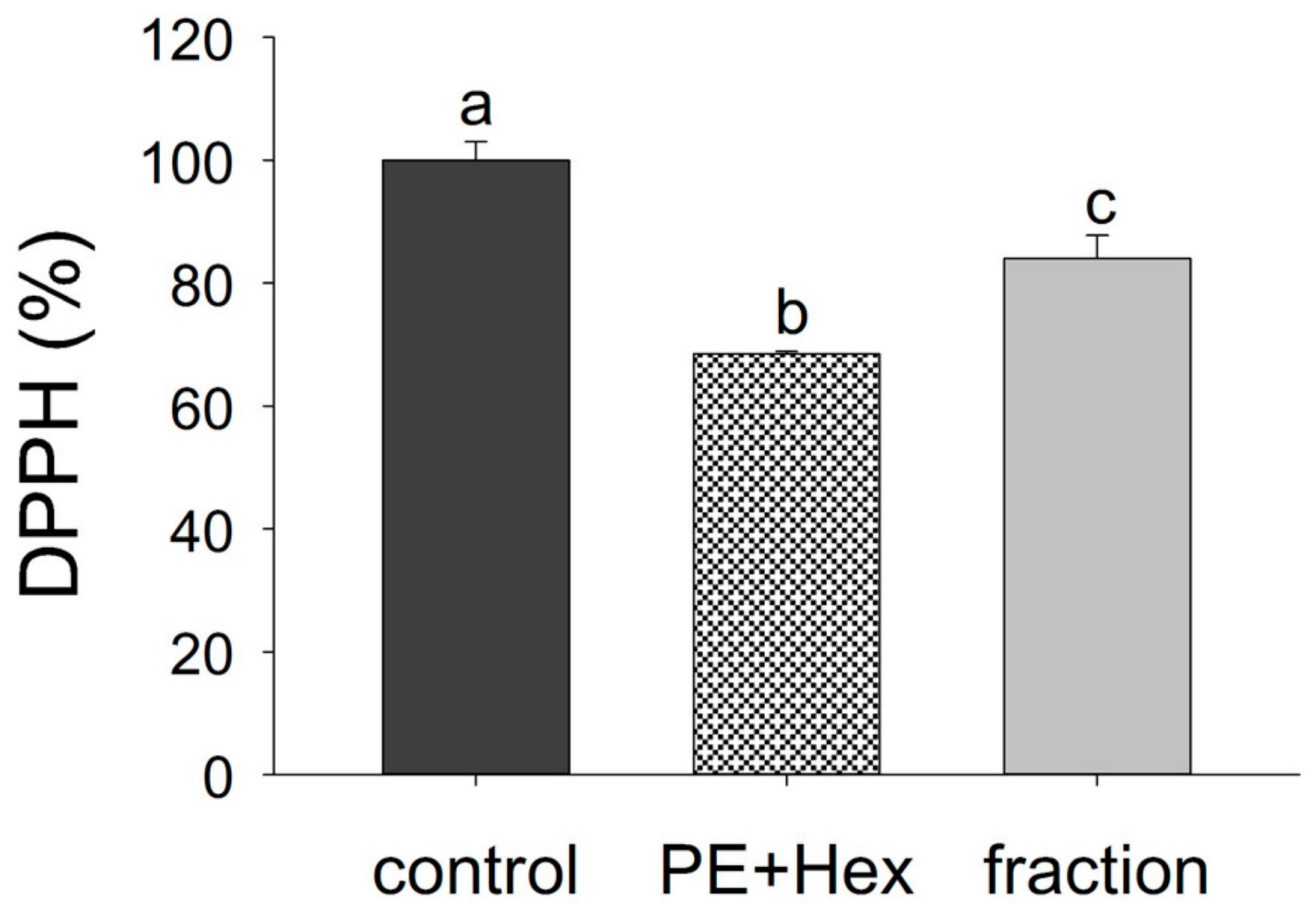
Free radical scavenging activity of the fraction containing 2-pentyl-3,5-dibutyl-dihydropyridine. The reduction of the DPPH signal (520 nm) was recorded for 60 min in the absence (control) and presence of PE (16:0/18:1) with hexanal and the collected fraction. Each experiment was performed thrice. Different letters indicate significant differences. Statistical analysis was performed applying one-way ANOVA with Holm–Sidak post hoc test.

To verify whether or not the radical scavenging activity of the mixture of PE (16:0/18:1) and hexanal was due to the carbonyl-amine adducts, such as the 2-pentyl-3,5-dibutyl-dihydropyridine, the fraction containing the adducts was isolated from the mixture. The reduction of the DPPH signal was recorded for 60 min in the presence and absence of PE (16:0/18:1) with hexanal or the collected fraction, containing 2-pentyl-3,5-dibutyl-dihydropyridine (Figure 5). The mixture of PE (16:0/18:1) and hexanal (31.5 ± 0.41%) had the strongest radical scavenging effect, followed by the collected fraction (16.0 ± 3.77%) and solvent alone (0.00 ± 2.79%) (*p* < 0.05). 

These results show that not only PE (16:0/18:1) with hexanal but also the fraction containing 2-pentyl-3,5-dibutyl-dihydropyridine had a radical scavenging effect. However, the collected fraction was not solely responsible for the radical scavenging activity of the mixture of PE (16:0/18:1) and hexanal. Other reaction products of the investigated mixture might contribute to the radical scavenging. It could be shown that pyrroles, formed during carbonyl-amine reactions, displayed different antioxidant effects [27,43]. Alkyl-substituted pyrroles with no free α-position had the highest antioxidant capacity, whereas pyrroles with a free α-position had negligible antioxidant effects. The presence of an oxygenated group further reduced the antioxidant effect [27]. However, in the current study we observed a radical scavenging effect, although one of the α-positions was not substituted, possibly explaining the low antioxidant capacity compared to antioxidants such as γ-tocopherol [9]. In addition, the hydrophilic or lipophilic properties of the pyrrole might influence the antioxidant activity as well. In tocopherol-added refined olive oil, a hydrophilic, lysine-based pyrrole had an antioxidant effect, whereas a lipophilic, PL-based pyrrole showed no effect [43]. Thus, many factors can influence the antioxidant activity of cyclic carbonyl-amine adducts. Structural differences in dihydropyridine derivatives, like the presence or absence of a hydrophobic side chain, might influence its radical scavenging activity as well. In the current study, some unknown carbonyl-amine adducts might contribute in addition to the dihydropyridine to the antioxidant effects of the mixture of PE (16:0/18:1) and hexanal. 

### 2.5. Tentative Identification of 2-Pentyl-3,5-Dibutyl-Dihydropyridine in Fortified Cold-Pressed Soybean Oils (CPSBO)

**Figure 6:**
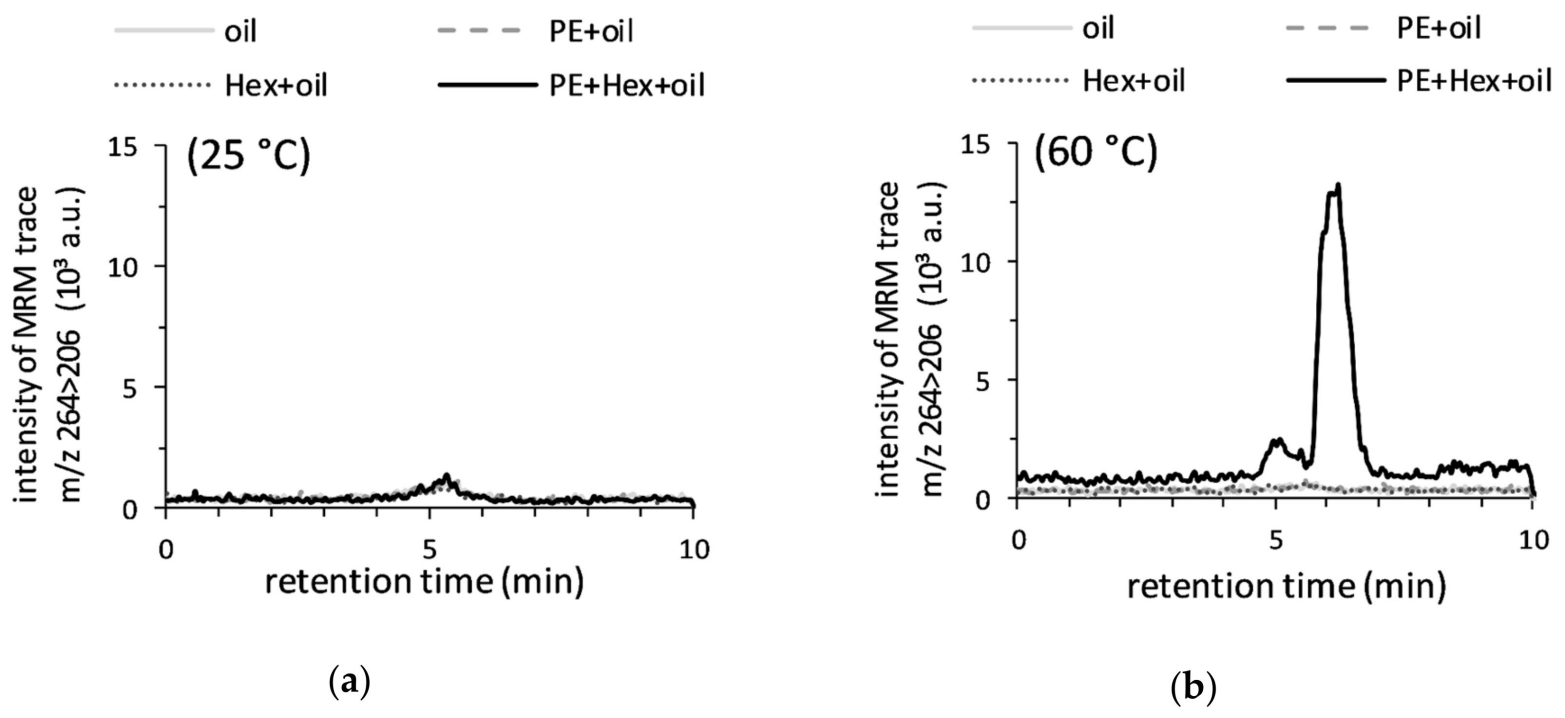
Formation of 2-pentyl-3,5-dibutyl-dihydropyridine in fortified cold-pressed soybean oils (CPSBO). A total of 50 µl of the oil samples fortified with 4 mM PE (16:0/18:1) and/or 8 mM hexanal was dissolved in 1 mL chloroform-methanol (2:1) prior to LC-MS/MS analysis. The overlaid MRM chromatograms represent the formation of the dihydropyridine-derivative (*m/z* 264 > 206) in oil after incubation with PE (16:0/18:1) and hexanal at 25 °C (**a**) or at 60 °C (**b**) for 60 min.

Since the antioxidant carbonyl-amine adduct was shown to be formed in the presence of PE (16:0/18:1) and hexanal in chloroform-methanol (2:1, *v/v*), its generation in PE (16:0/18:1) and hexanal fortified CPSBO, which represents oxidized cold-pressed oils, was investigated. CPSBO samples, dissolved in chloroform-methanol (2:1), were analyzed by LC-MS/MS. No peaks at the MRM trace *m/z* 264 > 206 were detected in CPSBO treated with PE (16:0/18:1) and hexanal at 25 °C for 60 min (Figure 6a). However, the carbonyl-amine adduct was present in CPSBO fortified with PE (16:0/18:1) and hexanal and incubated at 60 °C for 60 min (Figure 6b). No peaks were detected in the non-fortified CPSBO, in CPSBO fortified with PE (16:0/18:1) or with hexanal. These results confirm that the formation of the identified compound was possible in oil in the presence of PE (16:0/18:1) and hexanal. The natural hexanal content potentially present in CPSBO seemed to be too low to form the dihydropyridine in CPSBO fortified with PE (16:0/18:1) within an hour. However, it is well known from previous studies that hexanal content increases upon incubation of vegetable oils at 60 °C for 10 min, yielding a concentration of approximately 8 mM [44], which was used in the current study to fortify soybean oil.

### 2.6. Estimation of Radical Scavenging Activity of Fortified CPSBO

**Figure 7:**
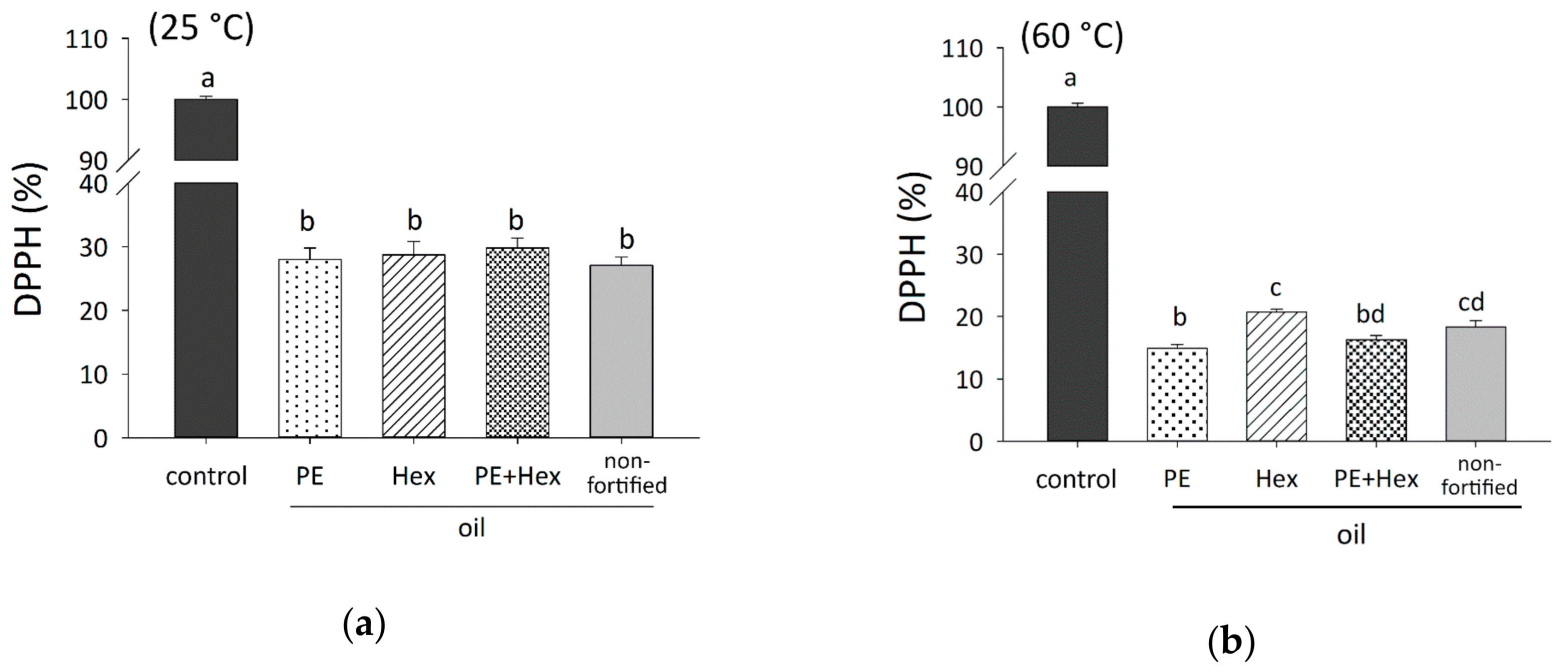
DPPH radical scavenging activity of the fortified and non-fortified oil samples. CPSBO (500 µl) was incubated with 4 mM PE (16:0/18:1) and/or 8 mM hexanal at 25 °C (**a**) and 60 °C (**b**) for 60 min. A total of 25 µl of the oil samples were dissolved in 1 mL toluene with DPPH (0.25 mM). In the toluene control, DPPH was set to 100% after 60 min. Values are shown as mean + SEM. Each experiment was performed thrice. Different letters indicate significant differences. Statistical analysis was performed using one-way ANOVA with Holm–Sidak post hoc test. control: non-fortified CPSBO.

The radical scavenging activity of the fortified and non-fortified CPSBO was measured 60 min after being combined with DPPH (0.25 mM). The DPPH signal (0.25 mM) was set to 100%. At 25 °C, the addition of fortified and non-fortified CPSBO to DPPH-containing toluene had a strong radical scavenging effect (Figure 7a). The addition of CPSBO, CPSBO with PE (16:0/18:1), CPSBO with hexanal and CPSBO with PE (16:0/18:1) and hexanal to DPPH-containing toluene increased the radical scavenging activity from 0.00% ± 0.50% to 72.9% ± 1.28%, 72.0% ± 1.81%, 71.2% ± 2.09% and to 71.2% ± 1.58%, respectively. However, no significant differences were observed between the fortified and non-fortified CPSBO samples. These results indicate that at 25 °C, the fortification of CPSBO with PE (16:0/18:1) and/or hexanal did not affect its radical scavenging activity. While a slight increase in the formation of the carbonyl-amine adduct (*m/z* 264 > 206) was obtained by the incubation of PE (16:0/18:1) with hexanal in chloroform-methanol (2:1) at 25 °C for 60 min, no peaks were observed in fortified CPSBO at 25 °C (Figure 6a).

The radical scavenging activity of approximately 70% in the fortified and non-fortified CPSBO samples can be explained by the presence of antioxidants, such as tocopherols. Crude soybean oil was shown to be rich in tocopherols (1670 ppm) with the following tocopherol ratio: γ (63.4%) > δ (31.1%) > α (4.6%) > β (0.7%) [12]. Furthermore, soybean oil contained carotenoids, with lutein and zeaxanthin being most abundant [45]. CPSBO, containing tocopherols and phenolic compounds, had a higher radical scavenging activity than refined oil [46]. The tocopherol content in soybean oil correlated with the reduction of DPPH [46].

The CPSBO samples, treated at 60 °C, decreased the intensity of the DPPH signal as well (Figure 7b). CPSBO fortified with PE (16:0/18:1) had the strongest radical scavenging activity (85.1% ± 0.62%), followed by CPSBO with PE (16:0/18:1) and hexanal (83.71% ± 0.67%), non-fortified CPSBO (81.67% ± 0.98%) and CPSBO with hexanal (79.29% ± 0.48%). We observed a significant difference between CPSBO with PE (16:0/18:1) and CPSBO with hexanal, as well as between CPSBO with PE (16:0/18:1) and non-fortified CPSBO. CPSBO fortified with the mixture of PE (16:0/18:1) and hexanal had a significantly higher radical scavenging activity than CPSBO with hexanal.

The LC-MS results showed that 2-pentyl-3,5-dibutyl-dihydropyridine was only detected in the CPSBO samples, which were fortified with PE (16:0/18:1) and hexanal and incubated for 60 min at 60 °C (Figure 6b). However, CPSBO fortified with PE (16:0/18:1) revealed the strongest radical scavenging activity with a significant difference to non-fortified CPSBO. In the solvent-based experiments, the mixture of PE (16:0/18:1) and hexanal had a stronger radical scavenging activity than the isolated fraction containing 2-pentyl-3,5-dibutyl-dihydropyridine (Figure 5). This indicates that various carbonyl-amine adducts might be generated simultaneously, influencing the radical scavenging activity. However, soybean oil is a far more complex system than chloroform-methanol (2:1) due to the presence of several minor components, such as free fatty acids, lipid oxidation products, tocopherols and metals [7,12]. Hence, various carbonyls, present as a result of lipid oxidation in the heated CPSBO, might have reacted with PE (16:0/18:1) generating several reaction products with a radical scavenging activity. In the mixture of PE (16:0/18:1) and hexanal, the abundance of hexanal might favor the formation of 2-pentyl-3,5-dibutyl-dihydropyridine and hinder the formation of other carbonyl-amine adducts with higher radical scavenging activities. Furthermore, although the addition of hexanal and PE (16:0/18:1) to CPSBO was able to demonstrate the formation of dihydropyridine in CPSBO, it might have impacted the ability of PE (16:0/18:1) to reduce the content of other carbonyls that were already present in the heated CPSBO. Hidalgo et al. [28] showed that the addition of slightly oxidized PE (200 ppm) to refined soybean oil had a higher antioxidant capacity than the presence of unoxidized PE by delaying the induction period of lipid oxidation. In the current study, the incubation of PE (16:0/18:1) with aldehydic lipid oxidation products, present in heated CPSBO (60 °C, 60 min), might have generated various carbonyl-amine adducts responsible for the radical scavenging effect in the DPPH assay. However, incubating CPSBO with PE (16:0/18:1) and hexanal at 25 °C for 60 min was not sufficient to produce 2-pentyl-3,5-dibutyl-dihydropyridine. This indicates that under these mild conditions, carbonyl-amine adducts might not have been formed yet in CPSBO or were not present in concentrations high enough to contribute to the radical scavenging activity. However, further oxidation of PE might decrease the shelf life of CPSBO, which indicates that two competing reactions are taking place simultaneously. On the one hand, the formation of antioxidant-producing carbonyl-amine reactions can occur, whilst, on the other hand, lipid oxidation products can be formed [28]. Furthermore, synergism between tocopherols and PLs was observed previously [19,23,43]. PE reduced the induction period of lipid oxidation in tocopherol-fortified olive oil (250 µg α-tocopherol/g), whereas PE did not show a protective effect in tocopherol-stripped oil [43]. In addition, PE (1000 µM) increased the lag phase of lipid hydroperoxides as well as hexanal in stripped soybean oil fortified with δ-, γ- or mixed tocopherols (100 µM). Enzymatically modified lecithin (2000–5000 µM), rich in PE, increased the lag phase of the hexanal formation in refined oil, which naturally contains tocopherols [47].Therefore, in the current study, PE (16:0/18:1) might also have a synergistic effect with the tocopherols present in CPSBO, treated at 60 °C for 60 min. However, the presence of aldehydes, such as hexanal, which represents advanced lipid oxidation, might favor the formation of carbonyl-amine adducts in CPSBO rich in PE (16:0/18:1), thereby reducing the content of aldehydes associated with off-flavors and increasing the free radical scavenging activity of cold pressed oils. In future studies, it should be investigated whether the carbonyl-amine adduct is formed during the storage of CPSBO rich in PE with the hexanal content that is naturally present in soybean oil. Further research is needed to identify other carbonyl-amine reaction products contributing to the radical scavenging activity in oil rich in PE (16:0/18:1).

In conclusion, carbonyl-amine reactions between PE (16:0/18:1) and hexanal led to the formation of adducts with radical scavenging activities. The fraction, containing 2-pentyl-3,5-dibutyl-dihydropyridine, showed a statistically significant but weak radical scavenging effect, however, also other carbonyl-amine adducts seem to contribute to the radical scavenging activity. In addition, the formation of 2-pentyl-3,5-dibutyl-dihydropyridine could be demonstrated in CPSBO fortified with PE (16:0/18:1) and hexanal, representative for oxidized cold pressed oils. Lipid oxidation in cold-pressed oils rich in PE might be limited due to (1) the reduction of the content of aldehydic off-flavors by undergoing carbonyl-amine reactions and (2) the radical scavenging activity of the carbonyl-amine adducts formed by the aldehydic lipid oxidation products and amino-phospholipids, naturally present in cold-pressed oils. Maillard-type reactions might play an essential role in protecting cold pressed oils from lipid oxidation during the oil extraction, processing and storage. Further studies are warranted to unravel the mechanisms underlying the formation of carbonyl-amine adducts and the structural identity of yet unknown carbonyl-amine adducts in cold pressed oils. In addition, lipid aldehydes and aminophospholipids might also form highly colored polymers through aldol condensation and carbonyl-amine polymerization. The role of these non-enzymatic browning reactions in preventing the deterioration of cold-pressed oils needs to be investigated in future studies. 

## 3. Materials and Methods 

### 3.1. Chemicals and Samples 

PE (16:0/18:1) in chloroform (25 mg PE/mL) was purchased from Avanti Polar Lipids, Inc. (Alabaster, AL, United States). Cold pressed soybean oil (CPSBO) was obtained from a local vendor (Hagenthaler Oelmanufaktur, St. Andrä-Wördern, Austria). All other chemicals were from Carl Roth (Karlsruhe, Germany) or Sigma-Aldrich (St. Louis, MO, USA). All solvents used as a mobile phase in liquid chromatography were LC-MS grade.

### 3.2. Preperation of the Solvent-Based Samples

PE (16:0/18:1) (0.8 mM), hexanal (1.6 mM) and a mixture of both compounds were dissolved in chloroform-methanol (2:1, *v/v*) and incubated in the dark under continuous stirring at 25, 40 and 60 °C for 0, 15, 30, 60 min, according to Stadelmann-Ingrand et al. [38]. The concentration of PE (16:0/18:1) is the representative concentration of PE (16:0/18:1) in crude soybean oil under the assumption that 2% of the total lipids are PLs [48], of which one fifth is PE [14], and of which approximately 10% is PE (16:0/18:1) [18]. As the hexanal concentration depends on the extent of lipid oxidation and cold-pressed oils are subjected to approximately 60 °C during seed conditioning and oil pressing, the maximum concentration of hexanal detected in vegetable oils incubated at 60 °C for 10 min was chosen [44]. The samples (0.5 mL) were prepared in 20 mL amber glass vials, which were coated with nitrogen and closed with gas tight caps. The samples were stored at −20 °C until analysis.

The samples prepared for the identification of the carbonyl-amine adducts by LC-ESI-QTOF-MS/MS were incubated at 40 °C for 21 h to achieve high yields of carbonyl-amine adducts.

### 3.3. Tenatative Identification of Carbonyl-Amine Adducts by LC-ESI-QTOF-MS/MS and NMR

The samples (10 µl) were injected into an UltiMate 3000 series HPLC-system (Dionex/Thermo Fisher Scientific, Germering, Germany). The samples were separated on an Atlantis C18 column (3 µm; 2.1 × 150 mm, Waters, Milford, MA, USA) with a flow rate of 0.3 mL/min at a column temperature of 25 °C. The mobile phase consisted of eluent A (acetonitrile/H_2_O 60:40 (*v/v*) containing 0.1% formic acid and 10 mM ammonium formate) and eluent B (acetonitrile/isopropanol 20:80 (*v/v*) with 0.1% formic acid and 10 mM ammonium formate). The HPLC-gradient was as follows: from 0 min to 10 min 30% B; 10 min to 18 min 30% B to 75% B, 18 to 22 min 75% B. 

The mass spectrometer (micrOTOF-Q II ESI-Qq-TOF, Bruker Daltonics, Bremen, Germany) wasoperated in the positive ion mode with the following parameters: capillary voltage 4.5 kV, nebulizer 1.2 bar (N_2_), dry gas flow 8.0 L/min (N_2_), dwell time < 5 s, dry temperature 180 °C. The scan range was recorded from *m/z* 50 to 2500. MS/MS experiments were performed with a collision energy of 15 eV. For the identification of the compound, [M+H]^+^ adducts were considered. In consecutive runs, a maximal *m/z* deviation of 10 ppm and a maximal peak width of 20 s was tolerated. The reference ions *m/z* 118.0862, 322.0481, 622.0289 and 922.0097 (Agilent ESI-L low concentration tuning mix, G1969-85000, Santa Clara, CA, United States) were used for mass axis calibration. 

^1^H NMR spectra were recorded on a Bruker Avance III 500 MHz spectrometer (Bruker Biospin GmbH, Rheinstetten, Germany). The spectra were referenced internally to chloroform resonances.

### 3.4. Time- and Temperature-Dependent LC-MS/MS Experiments

The samples were injected (5 µl) into a LCMS-8040 system (Shimadzu, Vienna, Austria) and separated on a C18 column (Kinetex EVO, 150 × 3.0 mm, 5 µm, Phenomenex, Aschaffenburg, Germany) with a column oven temperature of 25 °C. The flow rate was set at 0.5 mL/min. The mobile phase was H_2_O with 0.1% formic acid (eluent A) and acetonitrile with 0.1% formic acid (eluent B). The HPLC-gradient was as follows: 0–25 min with 30% to 50% B, 25–27 min with 50% to 100% B.

The tandem MS was operated in the positive ion mode with an ESI source. The dwell time was set to 100 ms, the nebulizing gas flow was 3 L/min and the drying gas flow was 12 L/min. The temperature of the desolvation line was 150 °C, the heat block temperature was 350 °C and argon was used as a CID gas. The samples were recorded in MRM mode with *m/z* 264 > 206, *m/z* 264.25 > 136 and 264 > 105. The peak with the highest intensity at *m/z* 264 > 206 was used for the comparative analysis.

### 3.5. Quality Procedures Applied to All MS/MSEexperiments

System suitability blanks, consisting of chloroform-methanol (2:1), and process blanks, where the solvent mixture was subjected to sample preparation, were considered at the beginning and the end of each analytical batch. Carry over effects could be excluded by including blank samples after each test sample injection. Pooled quality control (QC) samples were generated from all solvent-based and oil samples to consider repeatability and condition the analytical platform. After the system suitability and process blanks, five QC samples were injected at the beginning of the batch. In addition, after every fifth test sample injection, one QC sample was analyzed.

### 3.6. Isolation of the Fraction Containing Carbonyl-Amine Adducts

The mixture of PE (16:0/18:1) and hexanal was prepared, as described above (3.2). The samples (100 µl) were injected into the Ultimate 3000 HPLC system and separated on a C18 column (Kinetex EVO, 150 × 4.6 mm, 5 µm, Phenomenex, Aschaffenburg, Germany) with a flow rate of 1.5 mL/min. The mobile phase consisted of eluent A (H_2_O with 0.1% formic acid) and eluent B (acetonitrile with 0.1% formic acid). The linear HPLC-gradient was as follows: from 0 to 10 min from 30% B to 40% B; from 10 to 12 min B increased to 100%; 100% B was maintained until 20 min. The eluent time of the identified compound was determined by MS. The fraction, collected at 4–5.7 min in 20 mL amber glass vials, was subjected to LC-MS and NMR for identification of the carbonyl-amine adduct and used to compare its radical scavenging activity with those of the mixture of PE (16:0/18:1) and hexanal. The solvent of the collected fraction was evaporated under nitrogen and the fraction was freeze-dried to eliminate water and acetonitrile.

### 3.7. Preparation of CPSBO Samples

CPSBO was centrifuged prior to the sample preparation. PE (16:0/18:1) (4 mM) and/or hexanal (8 mM) were dissolved in CPSBO (500 µl). The fortified CPSBO samples and non-fortified CPSBO were incubated under nitrogen and continuous stirring in the dark in amber glass vials with gas tight caps at 25 or 60 °C for 60 min, as described above. A total of 50 µl of the samples were dissolved in 1 mL chloroform-methanol (2:1) and stored under nitrogen at −20 °C until analysis with LC-MS.

### 3.8. LC-MS Analysis of CPSBO Samples

The samples (10 µl) were injected into the HPLC with the same gradient as described above for the time- and temperature-dependent experiments. Only the eluent of the first 10 min of the run was directed into the MS for analysis since the compound of interest eluted at 6.4 min. The rest of the run was collected as waste to avoid the accumulation of non-polar compounds, such as triglycerides of CPSBO, into the MS-system. All other settings of the instrument and the mobile phase were as described above. 

### 3.9. Estimation of Radical Scavenging Activity of Samples

The solvent-based samples were incubated at 25 and 60 °C for 60 min, under the same conditions as described above (3.2). The samples were transferred into Eppendorf tubes and the solvent (chloroform-methanol (2:1)) was evaporated under nitrogen. The dried samples were redissolved in toluene. For photometrical analysis, 0.1 mM of the stable radical, 2,2-diphenyl-1-picrylhydrazyl (DPPH), was added to the mixture of 0.5 mM PE (16:0/18:1) and 1 mM hexanal. The absorbance of DPPH (0.1 mM) was determined at 520 nm on a Tecan reader (infinite 200, Tecan Group Ltd, Männedorf, Switzerland) after 60 min and compared with the absorbance of DPPH (0.1 mM) in the presence of PE (16:0/18:1) (0.5 mM), PE (16:0/18:1) with hexanal (0.5 + 1 mM) and hexanal (1 mM). A total of 100 µl samples were transferred onto a 96-well plate with a lid and placed into the Tecan reader. The absorbance of toluene was subtracted from each measurement. The mean of the absorbance of the DPPH solution (${\bar{X}}_{DPPH}$) was set to 100% and was compared to the absorbance of the remaining DPPH of each sample (*X_sample_*). The DPPH (%) content of each sample was calculated as followed:

(1) $$DPPH\;\left(\%\right)=\;\left(100\;\times \;X_{sample}\right)/\;{\bar{X}}_{DPPH}\;.$$

The radical scavenging activity of the CPSBO samples fortified with 4 mM PE and 8 mM hexanal was determined as described above. However, 25 µl of the CPSBO samples were dissolved in 1 mL toluene containing 0.25 mM DPPH. The samples were transferred onto a 96-well plate and placed into the Tecan reader.

To determine the radical scavenging activity of the fraction containing the carbonyl-amine adduct, the dried sample was redissolved in toluene. The decrease in absorbance of DPPH within 60 min was calculated for each sample and compared with the absorbance of DPPH as described for the solvent-based samples. The concentration of the carbonyl-amine adduct in the fraction was the same as in the solvent-based samples with 0.5 mM PE and 1 mM hexanal. The radical scavenging activity of the mixture of PE (16:0/18:1) and hexanal was compared with the DPPH reducing capacity of the fraction.

### 3.10. Statistical Analysis

Sigma Plot 13.0 was used for statistical analysis at a five percent significant level. The results are presented as mean ± SEM. The data of the time and temperature dependent experiments (*n* = 5) were normalized via natural log-transformation in Excel 2016 and were investigated with two-way analysis of variance (ANOVA) in Sigma Plot 13.0. One-way ANOVA was used to analyze the radical scavenging activity (*n* = 3). The Holm–Sidak test was applied as a post-hoc test in all analysis since it can be used for pairwise comparisons, relevant for studying the time- and temperature-dependent formation of the carbonyl-amine adduct as well as the free radical scavenging activity of the different samples. It is known to be more powerful than Tukey and Bonferroni tests for detecting significant differences.

## References

[1] Mozaffarian D., Micha R., Wallace S. (2010). Effects on coronary heart disease of increasing polyunsaturated fat in place of saturated fat: A systematic review and meta-analysis of randomized controlled trials. PLoS Med..

[2] Imamura F., Micha R., Wu J.H.Y., de Oliveira O.M.C., Otite F.O., Abioye A.I., Mozaffarian D. (2016). Effects of saturated fat, polyunsaturated fat, monounsaturated fat, and carbohydrate on glucose-insulin homeostasis: a systematic review and meta-analysis of randomised controlled feeding trials. PLoS Med..

[3] Doorn J.A., Petersen D.R. (2002). Covalent modification of amino acid nucleophiles by the lipid peroxidation products 4-hydroxy-2-nonenal and 4-oxo-2-nonenal. Chem. Res. Toxicol..

[4] Grosshagauer S., Steinschaden R., Pignitter M. (2019). Strategies to increase the oxidative stability of cold pressed oils. LWT Food Sci. Technol..

[5] Mei W.S.C., Ismail A., Esa N.M., Akowuah G.A., Wai H.C., Seng Y.H. (2014). The Effectiveness of Rambutan (*Nephelium lappaceum* L.) Extract in stabilization of sunflower oil under accelerated conditions. Antioxidants.

[6] Pignitter M., Stolze K., Gartner S., Dumhart B., Stoll C., Steiger G., Kraemer K., Somoza V. (2014). Cold fluorescent light as major inducer of lipid oxidation in soybean oil stored at household conditions for eight weeks. J. Agric. Food Chem..

[7] Steenson D.F., Lee J.H., Min D.B. (2002). Solid phase microextraction of volatile soybean oil and corn oil compounds. J. Food Sci..

[8] Schieberle P., Grosch W. (1981). Model experiments about the formation of volatile carbonyl compounds. J. Am. Oil Chem. Soc..

[9] Zaunschirm M., Pignitter M., Kienesberger J., Hernler N., Riegger C., Eggersdorfer M., Somoza V. (2018). Contribution of the ratio of tocopherol homologs to the oxidative stability of commercial vegetable oils. Molecules.

[10] Jeleń H.H., Obuchowska M., Zawirska W.R., Wa̧sowicz E. (2000). Headspace solid-phase microextraction use for the characterization of volatile compounds in vegetable oils of different sensory quality. J. Agric. Food Chem..

[11] Warner K., Evans C.D., List G.R., Dupuy H.P., Wadsworth J.I., Goheen G.E. (1978). Flavor score correlation with pentanal and hexanal contents of vegetable oil. J. Am. Oil Chem. Soc..

[12] Jung M.Y., Yoon S.H., Mln D.B. (1989). Effects of processing steps on the contents of minor compounds and oxidation of soybean oil. J. Am. Oil Chem. Soc..

[13] Verleyen T., Sosinska U., Ioannidou S., Verhe R., Dewettinck K., Huyghebaert A., De Greyt W. (2002). Influence of the vegetable oil refining process on free and esterified sterols. J. Am. Oil Chem. Soc..

[14] Chapman G.W. (1980). A Conversion factor to determine phospholipid content in soybean and sunflower crude oils. J. Am. Oil Chem. Soc..

[15] Mounts T.L., Abidi S.L., Rennick K.A. (1996). Effect of genetic modification on the content and composition of bioactive constituents in soybean oil. J. Am. Oil Chem. Soc..

[16] Zhou L., Le Grandois J., Marchioni E., Zhao M., Ennahar S., Bindler F. (2010). Improvement of total lipid and glycerophospholipid recoveries from various food matrices using pressurized liquid extraction. J. Agric. Food Chem..

[17] O’Donnell V.B. (2011). Mass spectrometry analysis of oxidized phosphatidylcholine and phosphatidylethanolamine. Biochim. Biophys. Acta.

[18] Zhou L., Zhao M., Ennahar S., Bindler F., Marchioni E. (2012). Determination of phosphatidylethanolamine molecular species in various food matrices by liquid chromatography-electrospray ionization-tandem mass spectrometry (LC-ESI-MS^2^). Anal. Bioanal. Chem..

[19] Cui L., McClements D.J., Decker E.A. (2015). Impact of phosphatidylethanolamine on the antioxidant activity of α-tocopherol and trolox in bulk oil. J. Agric. Food Chem..

[20] Lee J., Choe E. (2009). Effects of phosphatidylcholine and phosphatidylethanolamine on the photooxidation of canola oil. J. Food Sci..

[21] Koprivnjak O., Škevin D., Valić S., Majetić V., Petričević S., Ljubenkov I. (2008). The antioxidant capacity and oxidative stability of virgin olive oil enriched with phospholipids. Food Chem..

[22] Cui L., Decker E.A. (2016). Phospholipids in foods: Prooxidants or antioxidants?. J. Sci. Food Agric..

[23] Judde A., Villeneuve P., Rossignol C.A., Le Guillou A. (2003). Antioxidant effect of soy lecithins on vegetable oil stability and their synergism with tocopherols. J. Am. Oil Chem. Soc..

[24] Márquez R.G., García M.M.D.C., Holgado F., Velasco J. (2014). Effectiveness of α-, γ- and δ-tocopherol in a CLA-rich oil. Antioxidants.

[25] Yoon S.H., Min D.B. (1987). Roles of phospholipids in flavor stability of soybean oil. Korean J. Food Sci. Technol..

[26] Dacaranhe C.D., Terao J. (2001). A unique antioxidant activity of phosphatidylserine on iron-induced lipid peroxidation of phospholipid bilayers. Lipids.

[27] Hidalgo F.J., Nogales F., Zamora R. (2003). Effect of the pyrrole polymerization mechanism on the antioxidative activity of nonenzymatic browning reactions. J. Agric. Food Chem..

[28] Hidalgo F.J., Nogales F., Zamora R. (2005). Changes produced in the antioxidative activity of phospholipids as a consequence of their oxidation. J. Agric. Food Chem..

[29] Zamora R., Hidalgo F.J. (2011). The Maillard reaction and lipid oxidation. Lipid Technol..

[30] Zamora R., Nogales F., Hidalgo F.J. (2005). Phospholipid oxidation and nonenzymatic browning development in phosphatidylethanolamine/ribose/lysine model systems. Eur. Food Res. Technol..

[31] Tsuji K., Kawai Y., Kato Y., Osawa T. (2003). Formation of *N*-(hexanoyl)ethanolamine, a novel phosphatidylethanolamine adduct, during the oxidation of erythrocyte membrane and low-density lipoprotein. Biochem. Biophys. Res. Commun..

[32] Zamora R., Hidalgo F.J. (2003). Phosphatidylethanolamine modification by oxidative stress product 4,5(E)-epoxy-2(E)-heptenal. Chem. Res. Toxicol..

[33] Mondaca N.B.A., Ávila V.L.A., González C.A.F., López C.J., Sánchez M.D.I., Campas B.O.N., Rodríguez R.R. (2017). Antioxidant and chelating capacity of Maillard reaction products in amino acid-sugar model systems: Applications for food processing. J. Sci. Food Agric..

[34] Mastrocola D., Munari M. (2000). Progress of the Maillard reaction and antioxidant action of Maillard reaction products in preheated model systems during storage. J. Agric. Food Chem..

[35] Hidalgo F.J., León M.M., Zamora R. (2006). Antioxidative activity of amino phospholipids and phospholipid/amino acid mixtures in edible oils as determined by the Rancimat method. J. Agric. Food Chem..

[36] Moore N.H. (1983). Oilseed handling and preparation prior to solvent extraction. J. Am. Oil Chem. Soc..

[37] Hwang S.S., Carlin J.T., Bao Y., Hartman G.J., Ho C.T. (1986). Characterization of volatile compounds generated from the reactions of aldehydes with ammonium sulfide. J. Agric. Food Chem..

[38] Stadelmann I.S., Pontcharraud R., Fauconneau B. (2004). Evidence for the reactivity of fatty aldehydes released from oxidized plasmalogens with phosphatidylethanolamine to form Schiff base adducts in rat brain homogenates. Chem. Phys. Lipids.

[39] Annibal A., Schubert K., Wagner U., Hoffmann R., Schiller J., Fedorova M. (2014). New covalent modifications of phosphatidylethanolamine by alkanals: Mass spectrometry based structural characterization and biological effects. J. Mass Spectrom..

[40] Israelachvili J.N., Mitchell D.J., Niham B.W. (1976). Theory of self-assembly of hydrocarbon amphiphiles into micelles and bilayers. J. Chem. Soc. Faraday Trans..

[41] Gupta R., Muralidhara H.S., Davis H.T. (2001). structure and phase behavior of phospholipid-based micelles in nonaqueous media. Langmuir.

[42] Hidalgo F.J., Zamora R. (2017). Chapter two-food processing antioxidants. Adv. Food Nutr. Res..

[43] Hidalgo F.J., León M.M., Zamora R. (2007). Effect of tocopherols in the antioxidative activity of oxidized lipid-amine reaction products. J. Agric. Food Chem..

[44] Van Ruth S.M., Roozen J.P., Jansen F.J.H.M. (2000). Aroma profiles of vegetable oils varying in fatty acid composition vs. concentrations of primary and secondary lipid oxidation products. Mol. Nutr. Food Res..

[45] Slavin M., Kenworthy W., Yu L.L. (2009). Antioxidant properties, phytochemical composition, and antiproliferative activity of Maryland-grown soybeans with colored seed coats. J. Agric. Food Chem..

[46] Kania M., Michalak M., Gogolewski M., Hoffmann A. (2004). Antioxidative potential of substances contained in cold pressed soybean oil and after each phase of the refining process. Acta Sci. Pol. Technol. Aliment..

[47] Xu N., Shanbhag A.G., Li B., Angkuratipakorn T., Decker E.A. (2019). Impact of phospholipid-tocopherol combinations and enzyme-modified lecithin on the oxidative stability of bulk oil. J. Agric. Food Chem..

[48] Nash A.M., Frankel E.N., Kwolek W.F. (1984). Degumming soybean oil from fresh and damaged beans with surface-active compounds. J. Am. Oil Chem. Soc..

